# Determination of Indoor Air Quality in Archives and Biodeterioration of the Documentary Heritage

**DOI:** 10.5402/2012/680598

**Published:** 2012-10-30

**Authors:** Sofía Borrego, Paola Lavin, Ivette Perdomo, Sandra Gómez de Saravia, Patricia Guiamet

**Affiliations:** ^1^Laboratory of Preventive Conservation, National Archive of the Republic of Cuba, Compostela 906 esquina San Isidro, Old Havana, P.O. Box 10100, Havana, Cuba; ^2^Departamento de Química, Instituto de Investigaciones Fisicoquímicas Teóricas y Aplicadas (INIFTA), Universidad Nacional de La Plata (UNLP), Casilla de Correo 16, Sucursal 4, 1900 La Plata, Argentina; ^3^CONICET, La Plata, Argentina; ^4^Facultad de Ciencias Naturales y Museo, UNLP, Casilla de Correo 16, Sucursal 4, 1900 La Plata, Argentina; ^5^CICBA, Argentina; ^6^Facultad de Ciencias Veterinarias, UNLP, Casilla de Correo 16, Sucursal 4, 1900 La Plata, Argentina

## Abstract

Documentary heritage is permanently subject to suffering from physical, chemical, and/or biological alterations. Biological deterioration by microorganisms (bacteria and fungi) causes undesirable changes on material properties. Microorganisms affect different organic, natural or synthetic substrates (cellulose, polycarbonates), metals, and compounds of optical and magnetic devices (CD, VHS). Paper made by vegetal fibers, functional additives (glue, optical polishers, consolidating agents), and inks with organic bindings are used as sources of nutrients. The environmental microorganisms that form the microbial charge of indoor air at repositories (archives, libraries) storing cultural heritage can deteriorate the different supports of heritage importance and affect human health as allergies and skin affections. The aims of this research were to study microbial contamination of the environment and its influence on biodeterioration by the biofilm formation and to analyze the relationship between environment microbiota and biofilm formation in materials stored at three archives in Argentina and in two repositories of the National Archive of the Republic of Cuba.

## 1. Introduction

Archives preserve documents written on papyrus, parchment papers, papers, and electronic supports. These organic, inorganic, and synthetic materials are deteriorated by physical, chemical, and biological agents [1]. 

As environmental microorganisms can deteriorate the different supports of heritage significance [2, 3] as well as to affect human health as allergies and skin affections [4], it is important to investigate the microbial concentration of indoor air at repositories (archives and libraries) to preserve the cultural heritage. These microorganisms can be carried by dust particles into the indoor archive repositories by means of the people and the air ventilation systems [5] and when settling on the document surface they create a microecosystem that stops the normal flux of air on them. This situation conditions the surfaces to absorb humidity that helps the microbial adherence and the subsequent biofilm formation [2, 6]. At the suitable temperature and relative humidity the air microbiota can coexist together with the collections and people in a specific ecosystem without causing significant damages. However, when increasing thermohygrometric values at the repository, microorganisms can accelerate biodeterioration [3]. In order to define the air quality at archive repositories and to know the indoor ecosystems, it is necessary to systematically evaluate the microbial concentration of the air [2]. 

Most of microorganisms exist at indoor environments are saprophytic, and they obtain nutrients for their metabolism from inorganic and organic material such as wood, paper, painting, dust, rocks, and so forth [2, 7]. Fungi are the most important organisms as biodeterioration agents of the organic material, they produce extracellular enzymes and their hyphae can exert mechanical pressure on the support causing weakness [8].

The development and maintenance of a fungal community on a shelf of a library and archive or in a single book or document depends on the spores that reach the surface of the material, on the microenvironment (temperature, relative humidity, light), on the water activity of the substrate, and on the events that help colonization of materials (insect dispersion, human contamination, external sources of fungal diversity). When considering paper stored in a closed environment, its colonization and biodegradation depends on species identity and composition since only cellulolytic organisms can exploit the bulk of the substrate. As in natural environments, the diversity-functioning relationship is driven by the presence or absence of key species by niche differentiation and species interaction [9]. 

The aims of this research were to study microbial air quality and its influence on biodeterioration by the biofilm formation and to analyze the relationship between environment microbiota and biofilm formation in materials stored in three archives in Argentina and in two repositories of the National Archive of the Republic of Cuba. 

## 2. Materials and Methods 

### 2.1. Characterization of the Archives

Studies were performed at Historical Archive of Museum of La Plata (HAMLP), Archive of Historical and Cartographic Research Department from the Geodesy Direction (AHCRD), Archive of Notaries of Buenos Aires Province (AN) located in La Plata city, Buenos Aires Province, Argentina; at two repositories: Photo Library (PL) and Map Library (ML) of the National Archive of the Republic of Cuba (NARC) situated in La Havana city. The temperature (*T*) and relative humidity (RH) were measured inside of these repositories at each point of sampling at the moment when the microbiological sampling was performed using a digital thermo hygrometer (Model 8705, Bresciani, Italy). 

### 2.2. Microbiological Sampling of Air

The determinations were made in October, 2010. The sedimentation method suggested by Omeliansky was used for the environmental microbiological sampling [10, 11]. Open Petri dishes at 2 m from the floor were placed for 30 minutes in five different points, by triplicate at the HAMLP, at the AHCRD, and at AN. Culture media employed were Nutrient Agar (Merck) for the bacteria growth and YGC (Yeast extract Agar, Glucose and Chloramphenicol, Merck) to isolate fungi. At the NARC repositories, the bacteria were isolated in the Agar Nutrient but the Malt Extract Agar (Merck) with addition of chloramphenicol (0.1%) [12]. Five different places were analyzed at the ML and two at the PL, by triplicate. Subsequently, the dishes were incubated at 28 ± 2°C for 72 hours and 7 days, respectively. 

Once dishes incubated, fungal and bacterial colonies were counted. Colony forming units per cubic metre (CFU · m^−3^) were determined, taking into account the following equation described by Omeliansky [10, 11]:

(1) $$\begin{array}{c} N=5a\cdot 10^{4}\;{\left(bt\right)}^{-1}, \end{array}$$

where *N*: microbial CFU · m^−3^ of indoor air; *a*: number of colonies per Petri dish; *b*: dish surface, cm^2^; *t*: exposure time, min.

relative microbial distribution was conducted according to Smith [13], where

(2) Relative  distribution   =Number  of  colonies  of  the  genus  or  speciesTotal  number  of  colonies  of  all  genera  or  species      ×100.

### 2.3. Documents Analyzed

All documents analyzed are from the 19th Century. At HAMLP three photos were analyzed, two paper photos (F1 and F2) and one glass slide (F3); at AHCRD, one book (L1) and one map (M3), both made of paper; at AN two paper notarial acts (P1a, P1b and P2). From the MLNARC two maps were analyzed, one of paper (M1) and the other of silk (M2) and from the PLNARC, two photographs, one of silk (F3A) and the other of paper (F4). 

### 2.4. Isolation of Microorganisms from Documents

Samples of 1 cm^2^ from the surface of the documents were taken using sterile cotton swabs [14, 15]. Cotton swabs were submerged in 1 mL of sterile distilled water and decimal dilutions were performed. Suitable dilutions of each sample were inoculated onto Nutrient Agar on Petri dishes to isolate total bacteria. They were incubated at 28 ± 2°C for 72 h and subsequently colonies were counted by spread plate [16]. Amylolytic and proteolytic bacteria were counted in differential culture media (Starch Agar and Frazier Gelatin Agar) and their percentages were determined in relation to the total aerobic bacteria. Acid-producing bacteria were enumerated in broth for total acidifying bacteria [6, 14]. 

A similar procedure was used to determine the absence/presence of sulphite reducing bacteria (*Clostridium *sp.), but the aliquots were inoculated into differential-reinforced *Clostridium* broth (DRCM) and incubated at (28 ± 2°C) for 15 days. In some cases cells were enumerated by the most probable number (MPN) method [16].

To count colonies of fungi, suitable dilutions of each sample from different materials were inoculated onto YGC Agar and incubated at 28 ± 2°C for 5 days prior to counting colonies [16].

### 2.5. Identification of the Microorganisms Isolated from Air and Documents

Cultural and morphological characteristics of fungal colonies were observed and the identification was performed according to different manuals [17–20]. Bacteria were typified according to the Gram stain and the biochemical tests described in the *Bergey's Manual of Systematic Bacteriology *[21]. *Bacillus* sp. was identified by molecular techniques [22].

### 2.6. Laboratory and SEM Studies

Microsamples from original documents were observed by scanning electron microscopy (SEM). *Bacillus* sp., a strain commonly isolated from documents, were tested. This microorganism was cultivated in tubes with solid mineral medium, and a strip of filter paper was placed as the sole carbon source. The same medium with the addition of 1% glucose was the control employed. The adhesions to paper together with biodeterioration were observed by SEM Jeol 6360 LV. For their observation, samples were prepared keeping them in a closed chamber with ethyl alcohol for 24 hours and metalized with Au/Pd.

### 2.7. Qualitative Determination of the Cellulolytic Activity and the Production of Pigments by Fungi

The fungal strains isolated were seeded in a culture medium whose saline composition for 1 L is sodium nitrate 2 g; dipotassium phosphate 1 g; magnesium sulphate 0,5 g; potassium chloride 0,5 g; yeast extract 0,5 g; ferrous sulphate 0,01 g; agar 20 g; pH = 5.5. A strip of filter paper 4,8 cm long and 1 cm wide (equivalent to 50 mg of filter paper) was used as the sole carbon source in one case and in the other, crystalline cellulose (1%), glucose (1%) was used as control. The cultures were incubated at 28 ± 2°C during 21 days [11, 23].

### 2.8. Qualitative Determination of Proteolytic Activities by Fungi

Proteolytic activity was determined using only gelatin hydrolysis assay in a tube test. In this case, each isolate was inoculated by puncture inside gelatin medium in a test tube. The medium composition was identical to that before assay, but gelatin at 120 g/L was added as the carbon source. The inoculated tubes were incubated for 7 days at 28 ± 2°C. Afterwards they were stored at 4°C and a gelatin hydrolysis reaction was evidenced by medium liquefaction when the tubes were inverted [11, 24].

### 2.9. Qualitative Determination of Amylolytic Activities by Fungi

Each fungal strain isolated was seeded in a Petri dish with a saline composition similar to the one previously used and starch was (5 g/L) employed as the carbon source. After 7 days of incubation at 28 ± 2°C, 5 mL of Lugol's reagent were added over each culture plate, and the presence of a colourless zone around the colonies was taken as an indication of the positive hydrolysis [11].

### 2.10. Determination of the Production of Acids by Fungi

A suspension of spores from the fungal strains isolated was seeded in a culture broth with a saline composition similar to the one previously used, but with glucose at 1%, pH was adjusted in 7. Cultures were incubated at the same temperature for three days and then the pH of the culture medium was measured using a pH meter [11]. 

### 2.11. Statistical Analysis

The ANOVA-1 and Duncan tests were used to compare the environment concentration of fungi and bacteria among different Argentinean archives. The Student's *t*-test was used to evaluate differences in the fungal and bacterial concentration of the air in two repositories of NARC. Results with *P* ≤ 0.05 were considered statistically significant.

## 3. Results 

When analyzing fungal and bacterial concentrations in the air of Argentinean archives (Table 1), a great variation in spite of having similar values of temperature and relative humidity can be observed. The Archive of the Notaries (AN) was that of greater total microbial concentration (14400 CFU · m^−3^) and where the greater fungi concentration in the environment was detected (7667 CFU · m^−3^).

Fungal and bacterial concentrations of the environment at the NARC repositories (Table 2), with a higher relative humidity, were lower than those obtained in some Argentinean archives. In these repositories fungal concentration oscillated between 60 and 261 CFU · m^−3^ and bacterial concentration ranged between 502 and 2149 CFU · m^−3^. However, the greatest fungal and bacterial concentrations were detected at the PLNARC.

**Figure 1:**
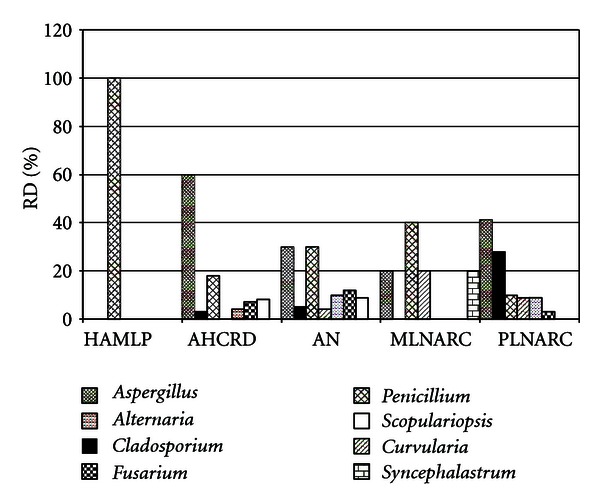
Relative distribution (RD) of the fungal genera at indoor air of Argentinean archives and NARC repositories.

The prevailing fungal genera isolated from air (Figure 1) were *Aspergillus* and *Penicillium*, although *Cladosporium, Curvularia, Alternaria, Fusarium, Scopulariopsis,* and *Syncephalastrum *were also isolated. At the AHCRD *Aspergillus *spp. prevailed (60%), at the AN, *Aspergillus* and *Penicillium* were isolated with the same relative distribution (30%), whereas at the HAMLP, colonies belonging to the *Penicillium* spp. were the only found (100%). At the MLNARC *Penicillium *prevailed (40%) whereas at the PLNARC it was *Aspergillus *the predominant one (41%). 

*Scopulariopsis *spp. was only found at the Argentinean archives of AHCRD and AN (8 and 9%, resp.) while *Syncephalastrum* spp. was only detected at MLNARC (20%). 

**Figure 2:**
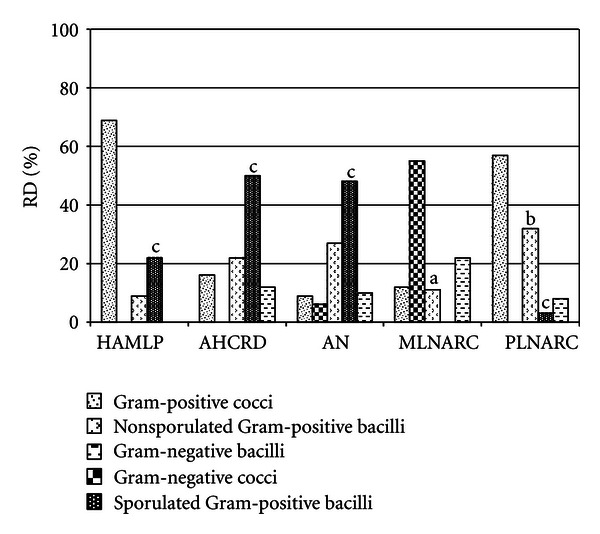
Relative distribution (RD) of different bacterial groups at indoor air of Argentinean archives and NARC repositories. (a) Indicates that 1% of the strains of the *Streptomyces* spp. genus is included. (b) Indicates that 8% of the strains of the *Streptomyces* spp. genus is included. (c) Indicate that these percentages belong to *Bacillus* genus.

In relation to bacterial groups of air (Figure 2), Gram-positive bacteria in the majority of archives studied except for MLNARC were Gram-negative bacteria prevailed (77%). In the air of the Argentinean archives the *Bacillus* spp. prevailed but it was not the case of the NARC, as only at the PLNARC a 3% of this bacterial genus was found. 

In air of NARC repositories low concentrations of *Streptomyces* spp. were detected, but this bacterial genus was not found in the air of Argentine archives.

**Figure 3:**
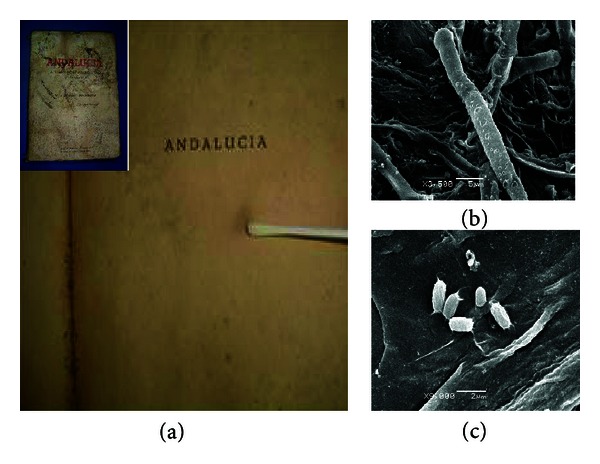
(a) Book with evidence of biodeterioration and (b) SEM micrograph showing biofilm formation in affected areas (1500×) (c) *Bacillus* sp. isolated from this document attached to filter paper (48 h of culture in laboratory)—extracellular polymeric substances (EPS) can be observed (9000×).

Microbial counts obtained in different documents analyzed (Table 3) showed a bacterial predominance regardless of the type of document support. This trend was similar to that obtained in the air from the repositories analyzed. In Figure 3 microbial adherence, biofilms formation, and extracellular polymeric substances (EPS) by scanning electron microscopy on document can be observed. It is worth mentioning that bacterial and fungal concentrations that adhered on the documents preserved at the AHCRD were significantly lower than the rest of the documents analyzed, regardless of the support they are made of. However, in some documents, the bacteria adhesion found was greater than that of fungi. It can also be observed that bacterial concentration of the map and photograph of silk (M2 and F3A) was significantly higher than in the rest of the maps and photographs of paper or crystal.

Physiological characteristics of bacteria isolated of documents showed high concentrations of amylolytic and proteolytic bacteria in great part of documents analyzed (Table 4). From the M2 map and the F3A photograph having a silk support, the greatest concentrations of proteolytic bacteria were isolated. Also, acidifying bacteria were isolated from the M3 paper map, F1 paper photograph and paper protocols P1a, P1b, and P2.

Sulphate reducing bacteria were not detected in the documents studied. *Clostridium* spp. were detected in F1 and F4 paper photographs, in F3A (silk photograph), and in P2 (paper protocol). 

Different fungal and bacterial genera were isolated from the documents (Table 5), the prevailing fungal *Aspergillus* spp. (*A. niger* and *A. flavus* were isolated from the great part of documents) and *Penicillium* spp. were detected in 83.3% and 50%, respectively. As regards M2 silk map, *Talaromyces helicus* Benjamín var. *major* (teleomorph of *Penicillium*) was isolated. Besides, from the M3 map and P1a protocol, both of paper, *Scopulariopsis* spp. was isolated. Cellulolytic, proteolytic and amylolytic activities, and production of acids were detected in all fungal strains isolated (Table 6).

In relation with bacteria isolated from documents, it was observed that 80% of them were Gram-positive bacteria such as *Bacillus*, *Clostridium,* and *Streptomyces* genera. Only 24% of Gram-negative bacteria were found.

## 4. Discussion 

When comparing environments of the archives, it can be seen that the HAMLP is the only archive having a microbial concentration lower than 150 CFU · m^−3^, the rest showed higher ones. Fungi concentrations were lower than 300 CFU · m^−3^ only at the HAMLP, but at the NARC (MLNARC and PLNARC) repositories, at the AHCRD and at the AN they were significantly higher, reaching a value of 7000 CFU · m^−3^.

Bacterial concentrations were higher than the fungal ones at AHCRD and NARC, whereas at the HAMLP and at AN the last ones were lower. 

In other previous samplings, when employing the sedimentation method proposed by Omeliansky, we obtained as a tendency that the fungal concentration was lower than the bacterial one [6, 11]; however, at the HAMLP and at AN it happened the contrary. This difference can be due to the fact that these archives do not have a ventilation system that facilitates water absorption by conidia and their sedimentation, as it was explained by Reponen et al. [25].

The low microbial concentrations at the HAMLP can be caused by the outdoor environment characteristics, which is much cleaner than the NARC. The NARC is located in a port zone, near factories and a busy avenue; consequently there is a high entry of dust into the deposits through the tubes of natural ventilation which do not have anti-dust filters. However, the AHCRD and AN archives, situated in the town center of La Plata, characterized by a high traffic and dust pollution, neither have ventilation systems nor suitable sanitary conditions. 

Despite the fact that there is no international standard to determine whether an indoor environment is contaminated or not, it has been suggested that environments with a microbial prevalence above 1000 CFU · m^−3^ should be considered contaminated [5]. Other authors consider that total microbial prevalence (bacteria and fungi combined) should not exceed 750 CFU · m^−3^—above this level the environment is regarded as contaminated [26], and still other authors consider that 300 CFU · m^−3^ should be the lower limit for fungi [27]. However, from 1998 the Ministery of Culture in Italy established that the air of a good quality in Italian archives, libraries, and museums cannot exceed the 750 CFU · m^−3^of bacteria or the 150 CFU · m^−3^ of fungi [28]. 

Considering what was said before it can be appreciated that the HAMLP and MLNARC indoor environments are not contaminated. In the PLNARC case the bacteria values are very high and for that reason it can be considered as a contaminated environment. The environments of the AHCRD and AN are highly contaminated. 

When analyzing the relationship between the microbial concentration and the *T* and RH values (Tables 1 and 2), it can be observed that there is a very high variability between Argentinean and Cuban archives. In the Argentinean archives, *T* and RH are suitable for document preservation [1] but the climatic conditions in the National Archive of Cuba are higher than optimal to preserve special materials (photographs and maps) despite being climatized the repositories. However, the adequate hygienic conditions of these repositories imply that concentrations of airborne microorganisms are lower than those of most Argentines archives. Similar results for the HAMLP and PLNARC have been obtained before [6, 11].

*Aspergillus *spp. was the predominant one in the air of PLNARC (41.3%) and in the AHCRD (40%) whereas *Penicillium* spp. predominated in the air of HAMLP (100%) and of the Map Library (40%). In the AN archive both genera were represented at similar proportions (30%). Other genera isolated were *Scopulariopsis*, *Fusarium*, *Alternaria*, *Curvularia, Syncephalastrum* and *Cladosporium*, in accordance with other authors [6, 7, 11, 12, 29].

It is known that the majority of the fungal genus isolated from the air of archives, libraries, and museums exhibit cellulolytic, proteolytic, and/or amylolytic activities, produce acids, excrete different pigments on the substrates (paper) (Table 6), and contribute to the formation of biofilms, which accelerate the deterioration of the different document substrates [2, 6, 11, 30]. When the relative humidity increases and indoor environments are without ventilation during long periods of time the conidia could be deposited more quickly over documents and deteriorate the document supports. 

In relation with environmental bacteria (cocci and rod shaped) the predominance of Gram-positives (HAMLP: 100%, AHCRD: 88%, AN: 84%, PLNARC: 92%) was observed. Only at MLNARC the Gram-negative bacteria predominate (77%). At the NARC deposits *Streptomyces *spp. strains were isolated (from 1 to 8%) considered as one of the most important genera in relation with occupational risks [31]. In all the Argentinean archives *Bacillus* spp. was the predominant genus according to similar results previously obtained [11]; however, this bacteria genus has been isolated in the environment of other NARC deposits [11, 29]. Others Gram-positive genera such as *Staphylococcus*, *Streptococcus *could be found and within the Gram-negative, strains of *Serratia *spp., *Pseudomonas* spp., *Serratia marcescens*,and *Enterobacter agglomerans* were identified. 

Genera as *Bacillus*, *Serratia*, *Staphylococcus*, *Streptococcus*, and *Streptomyces *were isolated by other authors in archive and museums indoor environment [7, 29, 32]. *Enterobacter *spp. has been isolated in other samplings in the NARC [11, 29].

Results obtained for the Gram-positive bacteria are in agreement with those reported by literature for this type of environments [33, 34].

Microbial adherence to document substrates showed viable bacteria and fungi. In particular, on the map (M2) and on the photo (F3A), both of silk, bacterial concentrations were higher than those obtained in other maps and photos, this could be due to the protein nature of support which would help the existence of a high concentration of viable proteolytic bacteria. Within the isolated Gram-positive bacteria genera *Bacillus*, *Clostridium *and *Streptomyces *could be found. Similar results were reported by Garside [35] and Michaelsen et al. [8].

*Bacillus* and *Streptomyces *genera are able to excrete hydrolytic enzymes such as proteases and chitinases [36, 37] that can degrade proteins and chitin that is part of the fungal wall [38]. It is probable that fungi concentrations on these documents (M2 and F3A) were the lowest ones. A bacterial genus anaerobic facultative, as *Clostridium* spp., was detected on the paper book (L1), the silk photograph (F3A) and the paper photograph (F4). This genus has also proteolytic and cellulolytic activities and seems to play an active role in the deterioration processes [8, 24, 30, 39], for these reason the strain could damage the document substrates if the weather conditions (T, RH, and ventilation) of the repositories environments would be favorable for its development. 

It was reported that during the manufacturing process of the paper photograph, the *Bacillus *spp. can pollute the gelatin belonging to the emulsion [40]. More recently, it was demonstrated that many bacteria can colonize the gelatin during the manufacturing process of the paper photograph, and despite of the fact that *Bacillus *was the predominant genus, nonsporulated bacteria from different species belonging to the *Salmonella*, *Kluyvera*, *Pseudomonas*, *Enterococcus*, *Streptococcus*, and *Staphylococcus* [41] genera that can liquefy gelatin were identified. 

This could explain the presence of other shapes of bacteria and Gram stain that is in accordance with every genus mentioned before. De Clerck et al. [42] reported that other endospore forming bacteria could also contaminate the gelatin, as they are able to resist the treatment employed during disinfection. This could justify the detection of *Bacillus* spp. and *Clostridium*, spp. strains on the photographs analyzed. 

*Bacillus* spp. has been already isolated from paper affected by foxing (rusty-red and irregular shaped areas caused by metal contamination, fungi and moisture condensation processes on paper) [2, 43] as well as from wooden art objects in museum environments [32]. Furthermore, *Bacillus* and related species have been shown to be the most commonly detected bacteria (up to about 20%) among the variety of microbial species isolated from the pulp and paper mill environment [44].

In addition, *Bacillus* spp. have been found as the predominant cellulolytic group of bacteria in landfill, where cellulose accounts for 40% to 50% of the municipal solid waste [45], and they form a significant proportion of the intestinal microbial community of soil invertebrates, especially among cellulose degraders [46].

The actinomycetes, in particular *Streptomyces*, are capable of decomposing relatively complex organic substances such as cellulose, pectin, chitin, proteins, and humic substances [47]. 

The presence of these bacteria on documents results to be highly risky for their preservation, they can degrade paper at a relative humidity of 90% in 24 hours what could be carried out if relative humidity of deposits would abruptly increase. 

In relation with fungi isolated from documents, the *Aspergillus* genus was the predominant and the *Aspergillus niger* and *Aspergillus flavus* species were detected in all documents, colonies of *Penicillium* spp., and *Cladosporium* spp. were isolated too. From the M2 silk map *Talaromyces helicus* Benjamín var. *major* (*Penicillium *teleomorph) was isolated [6] and in other documents colonies of *Alternaria, Scopulariosis* and nonsporing isolations were obtained. 

It is worth mentioning that the *Aspergillus* spp., *Penicillium *spp.*, Cladosporium *spp*., Alternaria *spp. and* Scopulariopsis *spp. were also isolated from the air of these rooms, which can colonize different surfaces due to their cosmopolitan distribution [8, 48]. It was found that teleomorphic shapes of fungi are difficult to isolate from the surface of art objects and documents [2], however, Michaelsen et al. [8] have recently detected teleomorphs from other fungi, showing that these fungal shapes can be isolated from documents. 

The succession of biological “events” that could have occurred to the object is somehow recorded in the microbial and fungal dead or living material present in it. Among the fungal species found in the documents, some of them could be considered strongly cellulolytic and, therefore, capable of colonizing pure cellulose. This is the case of the *Penicillium* teleomorph (*Talaromyces helicus* Benjamín var. *major*) which has cellulolytic activity, the *A. niger *y *A. flavus* species [6, 29, 49], *Penicillium *spp.*, Cladosporium *spp*., Alternaria* spp., and *Scopulariopsis* spp. [6, 29, 50].

## Figures and Tables

**Table 1:** Microbial air concentration obtained at Argentinean archives.

Archives	CFU · m^−3^				
	Fungi	Bacteria	Total microorganisms*	*T* (°C)	RH (%)
HAMLP	120^a^	100^d^	220	19.7 ± 1.4	50.6 ± 5.1
AHCRD	1271^b^	1422^e^	2693	18.7 ± 1.6	50.1 ± 5.2
AN	7667^c^	6827^f^	14494	18.5 ± 1.9	68.6 ± 1.7

^a,b,c,d,e,f^Indicates significant differences according to the Duncan test (*P *≤ 0.05) on comparing the environmental concentration of fungi and bacteria among different Argentinean archives. The microbial determination was made in 5 points by triplicate and the data averaged (*n* = 45). *Indicates the sum of the median concentration of fungi and bacteria.

**Table 2:** Microbial air concentration obtained at the repositories of the National Archive of the Republic of Cuba (NARC).

Repositories	CFU · m^−3^				
	Fungi	Bacteria	Total microorganisms*	*T* (°C)	RH (%)
Map library (ML)	60^a^	502^c^	562	24.0 ± 0.1	52 ± 1.0
Photo library (PL)	261^b^	1149^d^	1410	28.0 ± 0.5	65 ± 1.0

^a,b,c,d^Indicates significant differences according to the Student's test (*P*≤ 0.05) on comparing the fungi and bacteria concentration between the repositories of NARC. The microbial determination was made in 5 and 2 points by triplicate, respectively and the data averaged (*n* = 21). *Indicates the sum of the median concentration of fungi and bacteria.

**Table 3:** Microbial counts in documents.

Type of document	Location	CFU · m^−2^	
		Bacteria	Fungi
Paper map 1 (M1)	Map Library (NARC)	3.5 × 10^4^	2.2 × 10^4^
Silk map 2 (M2)	Map Library (NARC)	7.1 × 10^5^	2.0 × 10^2^
Paper map 3 (M3)	AHCRD	20	3
Paper book 1 (L1)	AHCRD	2	1
Paper photograph 1 (F1)	HAMLP	2.2 × 10^3^	1.0 × 10^2^
Paper photograph 2 (F2)	HAMLP	3.7 × 10^4^	—
Glass slide 3 (F3)	HAMLP	3.0 × 10^4^	1.0 × 10^3^
Silk photograph 3A (F3A)	Photo Library (NARC)	1.2 × 10^5^	1.5 × 10^2^
Paper photograph 4 (F4)	Photo Library (NARC)	3.9 × 10^4^	2.8 × 10^4^
Paper protocol 1a (P1a)	AN	1.3 × 10^3^	1.4 × 10^3^
Paper protocol 1b (P1b)	AN	4.0 × 10^2^	5.0 × 10^2^
Paper protocol 2 (P2)	AN	1.1 × 10^6^	2.0 × 10^4^

**Table 4:** Prevalence of bacteria isolated on documents exhibiting different physiological characteristic.

Type of document	(CFU · cm^−2^)				
	TAB	AB	PB	Acidifying B	Sulphite RB (*Clostridium* sp.)
M1	3.5 × 10^4^	1.1 × 10^4^	4.2 × 10^3^	−	−
M2	7.1 × 10^5^	3.0 × 10^4^	3.0 × 10^5^	−	−
M3	20	—	3	+	−
L1	2	—	—	−	−
F1	2.2 × 10^3^	3.4 × 10^3^	2.3 × 10^3^	+	+
F2	3.0 × 10^4^	1.0 × 10	3.7 × 10^4^	−	−
F3	3.0 × 10^4^	2.5 × 10^4^	3.0 × 10^4^	−	−
F3A	1.2 × 10^5^	7.2 × 10^3^	2.4 × 10^5^	−	+
F4	3.9 × 10^4^	—	1.6 × 10^4^	−	+
P1a	1.3 × 10^3^	—	20	+	−
P1b	4.0 × 10^2^	3	3	+	−
P2	1.1 × 10^6^	3 × 10^3^	1.1 × 10^3^	+	+

TAB: total aerobic bacteria determined on nutrient agar; AB: amylolytic bacteria determined on starch agar; PB: proteolytic bacteria determined on Frazier gelatin agar; Acidifying B: acidifying bacteria determined on broth for total acidifying bacteria; sulphite RB: sulphite reducing bacteria determined on reinforced *Clostridium* medium (DRCM).

**Table 5:** Type of microorganisms isolated from the different documents.

Type of microorganism and/or morphological characteristics	M1	M2	M3	L1	F1	F2	F3	F3A	F4	P1a	P1b	P2
Fungi												
*Aspergillus* spp.	+	+	+	+	+	−	−	+	+	+	+	+
*Penicillium* spp.	−	+	−	−	+	−	+	−	+	+	+	−
*Talaromyces helicus *	−	+	−	−	−	−	−	−	−	−	−	−
*Cladosporium *spp.	−	−	−	−	−	−	−	−	−	−	−	+
*Alternaria *spp.	−	−	+	−	−	−	−	−	−	−	−	−
*Scopulariopsis *spp.	−	−	+	−	−	−	−	−	−	+	−	−
Non-sporing isolated	−	−	−	−	−	−	+	−	−	−	−	−

Bacteria												
*Bacillus* spp.	−	+	+	−	+	+	+	+	+	+	+	+
*Clostridium *spp*. *	−	−	−	−	+	−	−	+	−	−	−	−
Gram-positive cocci	+	+	−	−	+	+	+	−	−	−	−	+
Gram-negative cocci	+	+	−	−	−	−	−	−	+	−	−	−
Non sporulated Gram-positive bacilli	−	+^a^	+	−	−	−	+	+	−	−	−	−
Gram-negative bacilli	−	+	−	−	+	−	−	+	+	−	−	−

^a^Indicates that strains of *Streptomyces* spp. were isolated too.

**Table 6:** Qualitative cellulolytic, proteolytic and amylolytic activities, production of pigments and acids by the fungi isolated from the air of the repositories studied and documents.

Isolate	Strain	Degradation of cellulose			Production of acids	Amylolytic Activity	Proteolytic Activity
		Growth on filter paper	Growth on crystalline cellulose	Pigment production^a^	pH	Starch degradation	Degradation of gelatin
AIR	*Aspergillus niger* 1	**+ + + **	**+ + +**	+	3.90	+	+
	*A. flavus *1	+ + +	+ +	−	5.99	+	+
	*A. clavatus *	±	−	−	5.20	+	+
	*A. versicolor *	+ +	+	+	6.10	+	+
	*A. terreus *	+ + +	+	−	4.46	+	+
	*Cladosporium cladosporoides *	+ + +	+ +	+	3.93	−	+
	*C. herbarum *	+	±	−	6.42	+	+
	*C. sphaerospermum *	+	±	−	6.47	+	+
	*C. oxysporum *1	+ +	+ +	+	6.90	−	−
	*C. elatum *	+ + +	+ + +	+	6.87	−	+
	*Cladosporium *spp.	+ + +	+ + +	+	6.70	+	−
	*Penicillium commune *1	+ + +	+	+	5.10	+	+
	*P. citrinum *1	−	−	−	5.26	+	+
	*P. chrysogenum *1	+ +	±	+	5.83	+	+
	*P. griseofulvum *	+ + +	+ +	+	5.73	+	+
	*Fusarium solani *	+ + +	+ +	+	5.95	+	−
	*Fusarium *sp.	+ +	+	+	5.32	+	+
	*Curvularia lunata *	+ +	±	+	6.77	+	−
	*C. pallescens *	+ + +	+	+	5.70	+	+
	*Alternariaalternata *	+ + +	+ +	+	6.54	+	−
	*Syncephalastrum *sp*. *	+ + +	+ + +	+	4.80	+	+

MAPS	*Aspergillus niger* 2	+ + +	+ + +	+	4.52	+	+
	*A. flavus *2	+ + +	+ + +	−	4.35	+	+
	*P. commune *2	+ + +	+ +	+	3.58	+	+
	*P. chrysogenum *2	+ + +	+	−	3.65	+	+
	*Alternaria *sp*. *	+ +	+	+	5.21	+	+
	*Talaromyces helicus *	+ + +	+	−	5.62	+	+

PHOTOS	*Aspergillus niger* 3	+ + +	+ + +	+	3.15	+	+
	*A. flavus *3	+ +	+	+	5.00	+	+
	*Penicillium decumbens *	+ + +	+	−	6.18	+	+
	*P. citrinum *2	+ + +	+ + +	−	3.14	+	+
	Non-sporing isolated	+ +	±	+	2.69	−	+

+ + +: Indicates abundant growth, + +: Indicates moderate growth, +: Indicates poor growth, it is also indicative of the presence of pigment, ±: Indicates very poor growth or production of pigment, −: Indicates NO growth and NO production of pigment. ^a^The production of pigments was evidenced on the filter paper strip.
